# BMI-Dependent Associations of the Estradiol-Linked Polymorphism rs117585797 of the *Anoctamin-2* Gene with Pre-Eclampsia

**DOI:** 10.3390/life16091472

**Published:** 2026-09-03

**Authors:** Maria Churnosova, Evgeny Reshetnikov, Inna Sorokina, Inna Aristova, Kirill Tsoy, Mikhail Voronin, Alexey Polonikov, Maria Solodilova, Mikhail Churnosov, Irina Ponomarenko

**Affiliations:** 1 Department of Medical Biological Disciplines, Belgorod State National Research University, 308015 Belgorod, Russia; churnosovamary@gmail.com (M.C.); reshetnikov@bsuedu.ru (E.R.); sorokina@bsuedu.ru (I.S.); aristova@bsuedu.ru (I.A.); tsoy@bsuedu.ru (K.T.); 142380@bsuedu.ru (M.V.); polonikov@rambler.ru (A.P.); solodilovama@kursksmu.net (M.S.); ponomarenko_i@bsuedu.ru (I.P.); 2 Department of Biology, Medical Genetics and Ecology, Kursk State Medical University, 305041 Kursk, Russia; 3 Research Institute for Genetic and Molecular Epidemiology, Kursk State Medical University, 305041 Kursk, Russia

**Keywords:** sex hormones, estradiol, pre-eclampsia, SNP, association

## Abstract

The aim of this study was to examine the modifying effect of body mass index (BMI) on the association of genome-wide association studies (GWAS)-significant for the level of sex hormones polymorphic loci with pre-eclampsia (PE). The work carried out genotyping of specially selected (according to data from previously conducted GWAS) 18 single nucleotide polymorphisms (SNP)that determine the content of various sex hormones in the organism. The sample of 891 pregnant women included two subgroups (they were formed based on the BMI values of women before pregnancy (pre-pregnancy BMI [preBMI]): preBMI < 25 (n = 592; 279 with PE/313 without PE) and preBMI ≥ 25 (n = 299; 152 with PE/147 without PE). The Firth’s penalized regression was used for the search of associations between SNPs and PE. As a whole, in the investigated cohort of women (PE [n = 431]/control group [n = 460], n = 891), an association of interactions of rs117585797 *anoctamin-2* × preBMI with PE was found: OR_A_ = 0.86, 95%CI_A_ = 0.76–0.96, p_permt_ = 0.020. The results of our associative analysis in the two cohorts under consideration indicated the presence of a significant association only among women with preBMI < 25: SNP rs117585797 *anoctamin-2* (C > A) was linked with PE risk (OR_A_ = 2.69, 95%CI_A_ = 1.26–5.72, p_permt_ = 0.011, power = 91.77%). In the women group with preBMI ≥ 25, we did not identify significant associations. The PE-associated locus rs117585797 of the *anoctamin-2* gene and 14 of its LD variants were functionally significant for 57 proteins involved in PE-significant biological pathways such as regulation of gene expression, hormone- and estrogen-dependent processes, embryonic development, and adipogenesis. In conclusion, this study is the first to demonstrate a BMI—specific correlation between GWAS-significant for estradiol levels polymorphism rs117585797 *anoctamin-2* (C > A) and PE: this SNP was PE-associated in preBMI < 25 pregnant women and not linked with PE risk in preBMI ≥ 25 pregnant women.

## 1. Introduction

Pre-eclampsia (PE) is a multisystem complication of pregnancy diagnosed in 5–8% of pregnant women [1,2]. PE is considered one of the main causes of maternal/perinatal morbidity/mortality, and it is associated with about 76,000 deaths among women and 500,000 deaths among infants annually [1]. Women with a history of PE have a significantly (almost 2-fold) increased risk of various cardiovascular diseases throughout their lifetime [3]. PE increases the risk of developing diseases of the nervous and cardiovascular systems, as well as diabetes, in the later life of a newborn [4].

PE is a complication of pregnancy with a pronounced genetic component (at least 60% of PE variability among Europeans is determined by genetic factors with the prevailing influence of the maternal genome [5,6]) and a significant role of a number of other risk factors (obesity, hypertension, diabetes mellitus, kidney disease, etc.) [1,7,8]. The impact of body mass index (BMI) on the risk of PE has been well documented in numerous experimental and clinical studies [7,8,9,10,11,12,13,14,15,16]. It is now well established that an increase in pre-pregnancy BMI (preBMI) leads to an increased risk of PE [13,14,15,16] due to such BMI-related mechanisms as endothelial dysfunction, chronic inflammation, insulin resistance, oxidative stress, dyslipidemia, the effect of leptin, an imbalance in the content of sex hormones, etc. [7,8,9,10,11,12,13,14,15,16,17,18,19].

Sex hormones (estrogens, progesterone, testosterone, etc.) are involved in the development of PE [20,21,22]. Their action is multi-vector and is aimed at regulating the processes of angiogenesis, the release of inflammatory/anti-inflammatory cytokines, oxidative stress, endothelial condition, regulation of uterine muscle tone, remodeling of spiral arteries, perfusion of the placenta, etc., all of which are important in the PE biology [20,21,22]. An imbalance in the content of sex hormones and their metabolites at certain stages of embryo/fetus development can lead to the formation of PE [20,21,22,23,24]. In turn, the level of sex hormones in a woman’s body largely correlates with BMI: an increase in BMI leads to an increase in concentrations of free/total estradiol, estrone, testosterone [25,26], and a decrease in BMI, conversely, causes the “normalization” of the level of sex hormones (a decrease in the estrogens/testosterone level) [27,28]. It is important to note that the content of sex hormones is genetically determined; there are numerous data from genome-wide association studies (GWAS) on polymorphic variants associated with the concentration of sex hormones [29,30,31,32,33,34,35,36,37,38,39,40,41,42,43].

Summarizing the above data, it should be noted that despite the numerous materials indicating the following significant links: (a) preBMI and PE [13,14,15,16,19]; (b) sex hormones (estrogens, testosterone, progesterone and others) and PE [18,21,22,44,45] (c) BMI and sex hormones [25,26,46,47]; (d) genetic polymorphisms and sex hormone levels (GWAS data) [29,30,31,32,33,34,35,36,37,38,39,40,41,42,43]; (e) genetic factors and PE [6,48,49,50,51,52,53,54,55] etc. (including GWAS-significant for BMI polymorphisms (for example, rs1421085 *FTO* [6])) genetic studies aimed at identifying links between GWAS-significant determinants of sex hormone levels and the PE risk under the modifying influence of BMI have not yet been conducted. The present study was designed to address this gap.

The aim of this study was to examine the modifying effect of BMI on the association of GWAS-significant for the level of sex hormones polymorphic loci with PE.

## 2. Methods

### 2.1. Study Subjects

At the planning stage of this study, using the Quanto program (version 1.2.4), we calculated the number of participants (PE/control) that we needed to include in each of the studied cohorts of pregnant women (BMI < 25; BMI ≥ 25) to obtain representative results (the study power was not less than 80%, α = 0.05, detection of differences in the frequencies of minor variants when comparing PE and control at the level of OR = 1.37–1.93 [additive model]). As a result of the calculations, it was revealed that the total number of participants in each of the cohorts of pregnant women under consideration (BMI < 25; BMI ≥ 25) must be ≥300 subjects.

**Figure 1:**
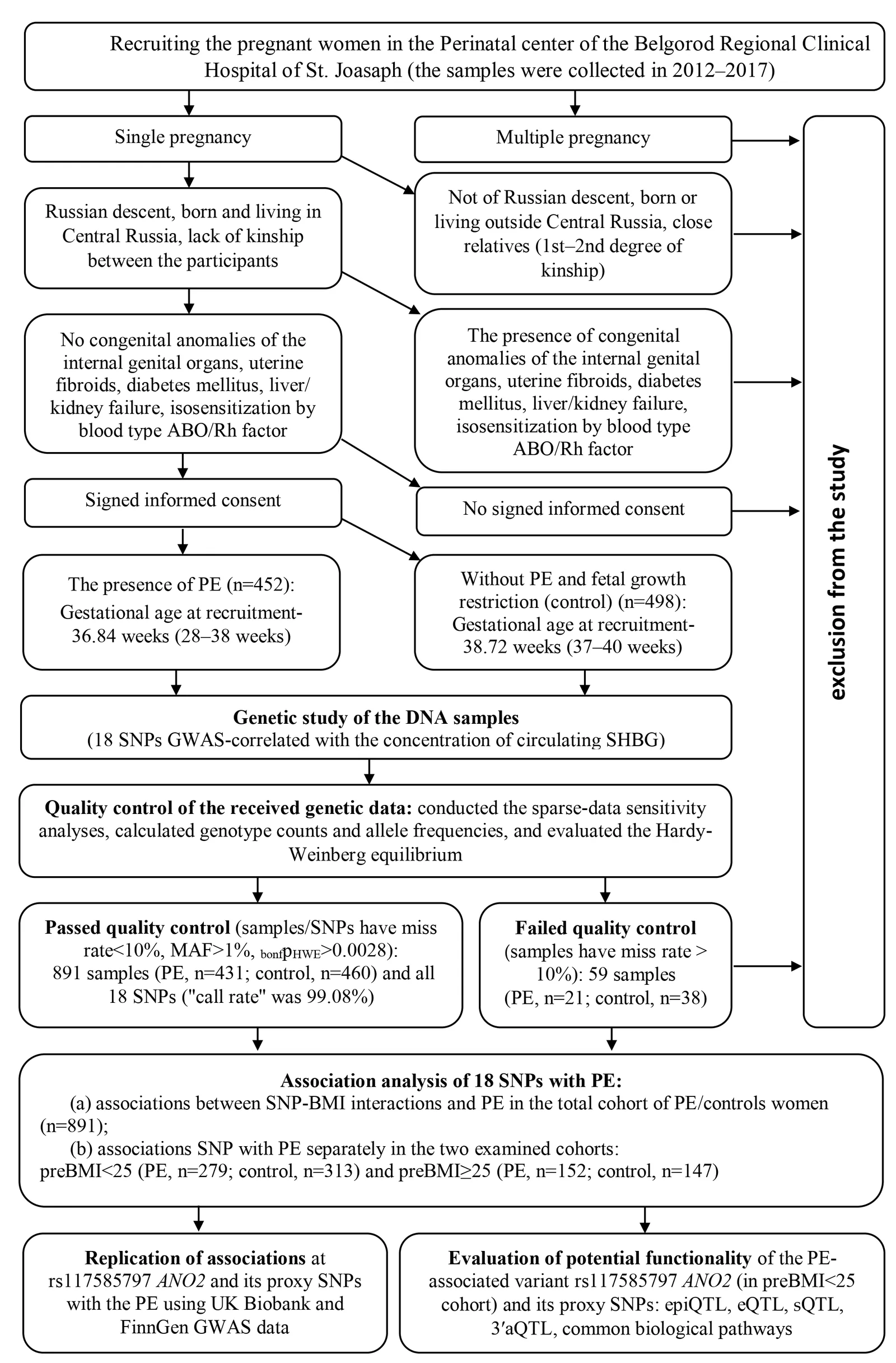
The general outline of the investigation.

The work was performed on a sample of 891 pregnant women (431 with PE/460 without PE [control]), among whom two cohorts were identified based on their BMI values before pregnancy (preBMI): preBMI < 25 (n = 592; 279 with PE/313 without PE) and preBMI ≥ 25 (n = 299; 152 with PE/147 without PE). The general outline of this study is shown in Figure 1. The following criteria were taken into account when diagnosing PE [56]: the presence of hypertension in a pregnant woman (systolic blood pressure (BP) 140 mmHg [and above] and/or diastolic BP 90 mmHg [and above]), which occurred after 20 weeks of pregnancy (with previously normal blood pressure) combined with either proteinuria (the total protein content/excretion in the urine [within 24 h] was not less than 300 mg [when present]) or utero-placental dysfunction or evidence of other maternal end-organ dysfunction. The PE diagnosis (and its absence during the formation of the control group) was performed by board-certified obstetricians at the specialized center [Perinatal Center] of the Belgorod Regional Clinical Hospital. The study groups (PE/without PE) included women who met the following criteria: (1) single pregnancy; (2) pregnant women without PE at the gestational age of delivery (37–40 weeks); women with PE at the time of treatment/delivery during hospitalization for PE (28–38 weeks); (3) self-reported Russian ethnicity; (4) were born/live in Central Russia [57]; (5) lack of relatives connections (1–2 degrees of kinship) between the participants. Pregnant women with a pathological placenta; uterine fibroids; malformations of the female genital organs; diabetes mellitus; severe somatic diseases accompanied by decompensation (liver/kidney failure), with isosensitization by blood groups ABO or Rh factor were excluded from the study [57,58]. In addition, pregnant women with fetal growth restriction were excluded from the control group. BMI parameters (kg/m^2^) were determined by the standard method [59] based on the height-weight data of the woman before pregnancy. Immediately before being included in the study, all women personally signed an informed consent document, previously approved by the Medical Ethics Committee of Belgorod State University. The phenotypic/clinical characteristics of the subjects as a whole (PE/control) are presented in Table 1. The detailed description of the two cohorts (preBMI < 25 [PE/without PE] and preBMI ≥ 25 [PE/without PE]) is given in Table 2; the same cohorts have been described in detail in our previous study [60].

### 2.2. DNA Examination (Selection/Genotyping of SNPs)

For the genetic study, DNA was used (previously obtained by us [60] from venous blood by phenol–chloroform extraction followed by ethanol precipitation), which was stored in low-temperature freezers, at a temperature of −80 degrees. When preparing DNA samples for genotyping, their quality was monitored: all DNA samples had the required degree of purity (the ratio of 260 nm/280 nm was in the range of 1.7–2.0) and concentration (10–20 ng/mL). All of the above measurements were performed on a NanoDrop™2000 spectrophotometer (Thermo Fisher Scientific, Waltham, MA, USA).

For this study, eighteen polymorphisms were selected according to the following criteria: (1) associations, according to previously conducted GWAS [29,30,31,32,33,34,35,36,37,38,39,40,41,42,43], with level of a number of sex hormones (estradiol, progesterone, testosterone (total, bioavailable), dehydroepiandrosterone sulfate (DHEAS)) and hormones that “regulate” their levels (globulin binding sex hormones (SHBG), luteinizing (LH) and follicle-stimulating (FSH) hormones), significant for the pathophysiology of PE [20,21,22] (Table S1); (2) the association with hormone phenotype in the female/predominantly female cohort (herewith the presence of such a link in the male group or in the combined male/female cohort was also taken into account) (Table S1); (3) the presence of GWAS-significant associations in European populations (Table S1); (4) the presence of potential impact functionality [61,62,63,64,65,66,67,68,69,70,71] (according to the data presented in HaploReg (accessed 5 July 2024) [72]) (Table S2); (5) the frequency of minor allele among Europeans is >1% (HaploReg data) (Table S2). So, using the above criteria, eighteen loci such as rs34670419 *ZKSCAN5* (progesterone, DHEAS, androgen metabolites), rs148982377 *ZNF789* (DHEAS, bioavailable testosterone, androgen metabolites), rs11031002 *FSHB* (LH, serum levels of protein CGA; FSHB), rs11031005 *FSHB* (FSH, testosterone (total, bioavailable), free androgen index (FAI) [testosterone/SHBG × 100]), rs112295236 *SLC22A10* (progesterone, progesterone metabolite, bioavailable testosterone), rs117585797 *ANO2* (estradiol), rs117145500 *CHD9* (FAI), rs17496332 *PRMT6* (SHBG), rs780093 *GCKR* (SHBG), rs10454142 *PPP1R21/KLRAQ* (SHBG), rs3779195 *BAIAP2L1* (SHBG), rs440837 *ZBTB10* (SHBG), rs7910927 *JMJD1C* (SHBG), rs4149056 *SLCO1B1* (testosterone (total, bioavailable), SHBG), rs8023580 *NR2F2* (total testosterone, SHBG), rs12150660 *SHBG* (total testosterone, SHBG), rs727428 *SHBG* (estradiol, progesterone, testosterone (total, bioavailable), SHBG), rs1641549 *TP53* (SHBG) were included in this study. Detailed information on the 18 loci selected for this study (source GWAS, population, hormone phenotype, effect allele, effect size) is presented in Table S1.

SNP genotyping was performed on a CFX96 device [73] with compulsory adherence of the genotyping quality control procedure (using positive/negative samples; repeated (blind) genotyping of every twentieth DNA sample [74]). The percentage of detected genotyping errors did not exceed 0.6%. The genotyping kits were specially designed/produced for the present study by the Russian R&D company TestGene (Ulyanovsk, Russia), specializing in the area of genetic research (https://testgene.com/).

### 2.3. Statistical Analysis

Before conducting the genetic statistical analysis, we performed the sparse-data sensitivity analyses, calculated genotype counts and minor allele frequencies (MAF), and checked the “compliance” of all considered loci of the Hardy-Weinberg law (HW_L_) in all four analyzed subgroups (preBMI < 25 [PE/control] and preBMI ≥ 25 [PE/control]) [75]. As a result, conducting the sparse-data sensitivity analyses, we excluded from further analysis SNPs/samples for which the proportion of missing values was >10%. Ultimately, after filtering/excluding of a number of samples (a total of 59) from the analysis, 891 samples (initially, the number of samples was 950) and all 11 polymorphic variants were included in the final genetic database for subsequent calculations (association search). The “call rate” for these samples/SNPs was 99.08%, which indicates sufficient quality of the received genetic data and allows it to be used in association analysis. Information about the genotype counts and MAF in the studied cohorts of pregnant women is provided in Table S3 (preBMI < 25 [PE/control]) and Table S4 (preBMI ≥ 25 [PE/control]). The MAF values for the examined loci exceeded the threshold value of 1% in all studied groups of women (preBMI < 25: PE [MAF ≥ 0.041]; control [MAF ≥ 0.014] (Table S3); preBMI ≥ 25: PE [MAF ≥ 0.020]; control [MAF ≥ 0.034] (Table S4)). An analysis of the investigated 18 loci distribution revealed their correspondence to HW_L_ among PE/control women in both cohorts under consideration (preBMI < 25: PE [0.098 ≤ p_HWl_ ≤ 1.000]; control [0.024 ≤ p_HWl_ ≤ 1.000] (Table S3); preBMI ≥ 25: PE [0.055 ≤ p_HWl_ ≤ 1.000]; control [0.029 ≤ p_HWl_ ≤ 1.000] (Table S4)] (the data were analyzed with Bonferroni adjustment, equal to the number of examined SNPs [n = 18], P_bonf_ > 0.0028).

At the beginning of this study, we analyzed the associations between SNP-BMI interactions (BMI was considered by us as a quantitative variable) and PE in the total cohort of women with PE (n = 431) and controls (n = 460). Association indicators (such generally accepted parameters as OR, 95%CI [76,77,78]) were calculated using logistic regression in the gPLink program (Java version 2.050) [79] using an additive genetic model (the most often used model in the association search [including in GWAS]). To solve the problem of multiple comparisons (we analyzed 18 SNPs), which leads to false positive results, we used permutation testing (as a result of adaptive permutation procedures, the p_permt_ index was calculated) [80,81]. Permutation testing, on the one hand, is a more “gentle” correction for multiple comparisons (as opposed to the Bonferroni correction), on the other hand, it is quite effective in solving this problem (multiple comparisons) even when studying large amounts of GWAS data without reducing the power of the study [80,81]. At this stage of the study, the results at p_permt_ ≤ 0.05 were considered statistically significant. The Bonferroni correction was not applied at this stage of the analysis, due to the fact that we considered only one comparison pair (PE-control) within the framework of one model (additive), and the number of loci under consideration (n = 18) was adjusted by adaptive permutation procedures.

At the next stage of this work, the indicators of the associations (OR, 95%CI) between SNPs and PE were obtained separately for each of the analyzed groups: females with preBMI < 25 and females with preBMI ≥ 25. All calculations were performed in a Java-adapted version of the gPLink program (PLINK v2.0.0-a.7.1) [79]. When calculating, we took into account the following points:

(a) the association analysis was done within the framework of a single genetic model, the additive model (the most frequently used model in the genetic association studies [including GWAS]), which does not require additional adjustments for multiple comparisons;
(b) the calculations were performed by Firth’s penalized regression (in connection with the analysis of a number of SNPs with rare minor alleles (Tables S3 and S4);
(c) to computation the association indicators in the two groups under consideration (preBMI < 25 and preBMI ≥ 25), a single list of covariates was used (in order to unify the list of covariates in the two analyzed groups); it included parameters for which differences between PE and control were found in at least one group (Table 2). The following parameters were used as covariates: age; pre-pregnancy BMI; family history of PE; number of spontaneous/induced abortions (data from Table 2).
(d) we also employed the first principal component (correction for population stratification) as a covariate (the distribution graph of the variances of the principal components [Scree plot] reaches a plateau after the first principal component);
(e) the prerequisite for our statistical analysis was the use of all possible procedures aimed at minimizing false-positive results, and for this purpose, firstly, we additionally introduced the Bonferroni correction (equal to 2) [82], taking into account the number of pairs being compared (preBMI < 25; preBMI ≥ 25). Secondly, we conducted adaptive permutation testing of the obtained associations [80,81]); as noted above, permutation testing provides an effective solution to the problem of multiple comparisons with a large number of SNPs (for example, GWAS data) (in our study, we considered 18 SNPs) and is a milder amendment than the Bonferroni correction [80,81].
(f) the power parameter of the received association was calculated (the Quanto program was used (ver.1.2.4, Los Angeles, CA, USA) [83]).

The problem of multiple comparisons at this stage of the work (examining associations of individual loci with PE in two comparison cohorts, preBMI < 25 and preBMI ≥ 25) was solved by introducing an additional Bonferroni correction (equal to the number of pairs of cohort compared, n = 2 (preBMI < 25; preBMI ≥ 25)), the use of permutation procedures (to adjust the number of 18 SNPs studied) and the examination of only one of the most widely accepted genetic model, additive. As a result of this stage of work, PE-SNP associations with values of p_permt_ ≤ 0.025/power ≥ 80% were considered statistically significant [60,84].

In order to confirm the association of rs117585797 *ANO2* with PE, established in our study, associations of this locus with PE and 14 SNPs in linkage disequilibrium (LD) with it [r^2^ ≥ 0.2, D′ = 0.94–1 (−1)] were replicated [85] using GWAS data from the UK Biobank (http://geneatlas.roslin.ed.ac.uk/; accessed on 14 July 2025) [86] and the FinnGen cohort (https://public-metaresults-fg-ukbb.finngen.fi/about; accessed on 15 July 2025) [87].

### 2.4. Predict Functions of PE-Linked SNPs

The estimated functionality of the PE-associated locus rs117585797 *ANO2* and 14 polymorphisms in linkage disequilibrium (LD) with it (r^2^ ≥ 0.20) was evaluated in silico using modern genetic resources on functional genomics such [88,89,90] as HaploReg (ver. 4.2, Cambridge, MA, USA, http://archive.broadinstitute.org/mammals/haploreg/haploreg.php accessed on 7 June 2025) [72], eQTLGen Consortium data (eQTLGen phase I) (https://www.eqtlgen.org/ accessed on 15 July 2025) [91], GTExproject (ver. 8, http://www.gtexportal.org/ accessed on 21 July 2025) [92], 3′aQTL-atlas (https://wlcb.oit.uci.edu/3aQTLatlas/ accessed on 28 July 2025) [93] and STRING https://string-db.org/ (accessed on 14 August 2025) [94].

## 3. Results

Overall, in the investigated cohort of women (PE [n = 431]/control group [n = 460], n = 891), an association of interaction of rs117585797 *anoctamin-2 (ANO2)* × preBMI with PE was found: OR_A_ = 0.86, 95%CI_A_ = 0.76–0.96,p_A_ = 0.019/p_permt_ = 0.020 [after permutation test] (Table 3).

The results of our association analysis in the two cohorts under consideration, preBMI < 25 and preBMI ≥ 25, indicate the presence of significant association only among women with preBMI < 25: polymorphic variant rs117585797 *ANO2* (C > A) is linked with PE risk (OR_A_ = 2.69, 95%CI_A_ = 1.26–5.72, p_A_ = 0.010/p_permt_ = 0.011, power = 91.77% [population frequency of PE—5%, the minor allele frequency in the preBMI < 25 cohort—2.72%, OR = 2.69, additive model, alpha = 0.05 for 2-sided test]) (Table 3). In the women group with preBMI ≥ 25, we did not identify significant associations (Table 3). There were also no significant associations between PE and the haplotypes examined (rs148982377 *ZNF789*-rs34670419 *ZKSCAN5* [7 chr, r^2^ = 0.67] and rs11031002-rs11031005 *FSHB* [11 chr, r^2^ = 0.74], p_permt_ > 0.025) (Table S5).

### 3.1. Replication of the Association at rs117585797 ANO2 and Proxy SNPs with the PE Risk Using UK Biobank and FinnGen GWAS Data

It is believed that the confirmation of the association identified in the study (association replication) allows to a certain extent to ensure that the “genotype–disease” relationship established in the work represents a reliable association, and is not a chance finding or artifact due to uncontrolled biases [95,96]. Therefore, we performed a replication analysis of the associations at rs117585797 *ANO2* and 14 LD polymorphisms with the PE risk using freely available UK Biobank and FinnGen GWAS data in women of European ancestry. The results this replication analysis are presented in Table 4.

Calculations performed using UK Biobank GWAS data for the “gestational hypertension/pre-eclampsia” phenotype revealed a significant association of rs118176536 *ANO2* with this phenotype (*p* = 0.037; p_HWE_ = 0.168; minor allele frequency (MAF) of the A allele = 0.021). It should be noted that according to the GeneATLAS (UK Biobank) data, the genome region under study, in which the PE-associated SNP rs117585797 *ANO2* and 14 LD polymorphisms were located (intron of the *ANO2* gene; chromosome 12:5981427-6011490 (GRCh37)) contains a number of other genetic variants associated with the “gestational hypertension/pre-eclampsia” phenotype: rs10774382 (*p* = 0.052; OR = 1.07), rs76418087 (*p* = 0.032; OR = 1.26), rs113445398 (*p* = 0.046; OR = 1.28).

Using FinnGen GWAS data, we obtained the following results: rs7296308 *ANO2* was associated with both the “pre-eclampsia or eclampsia” (*p* = 0.033) and “pregnancy hypertension” phenotypes (*p* = 0.044), and rs4764581 *ANO2* was correlated with the “pregnancy hypertension” phenotype (*p* = 0.048) (Table 4). It should be noted that according to the data of the FinnGen project, in the region of the *ANO2* gene there are two more SNPs associated with the both “pre-eclampsia or eclampsia” and “pre-eclampsia” phenotypes: rs574228461 (*p* = 0.00009; β = 0.45 and *p* = 0.0004; β = 0.43 respectively) and rs188451963 (*p* = 0.0001; β = −0.61 and *p* = 0.003; β = −0.44 appropriately). Two polymorphisms in the *ANO2* gene region were also associated with the “pregnancy hypertension” phenotype: rs574228461 (*p* = 0.039; β = 0.19) and rs184510890 (*p* = 0.001; β = −0.64).

**Figure 2:**
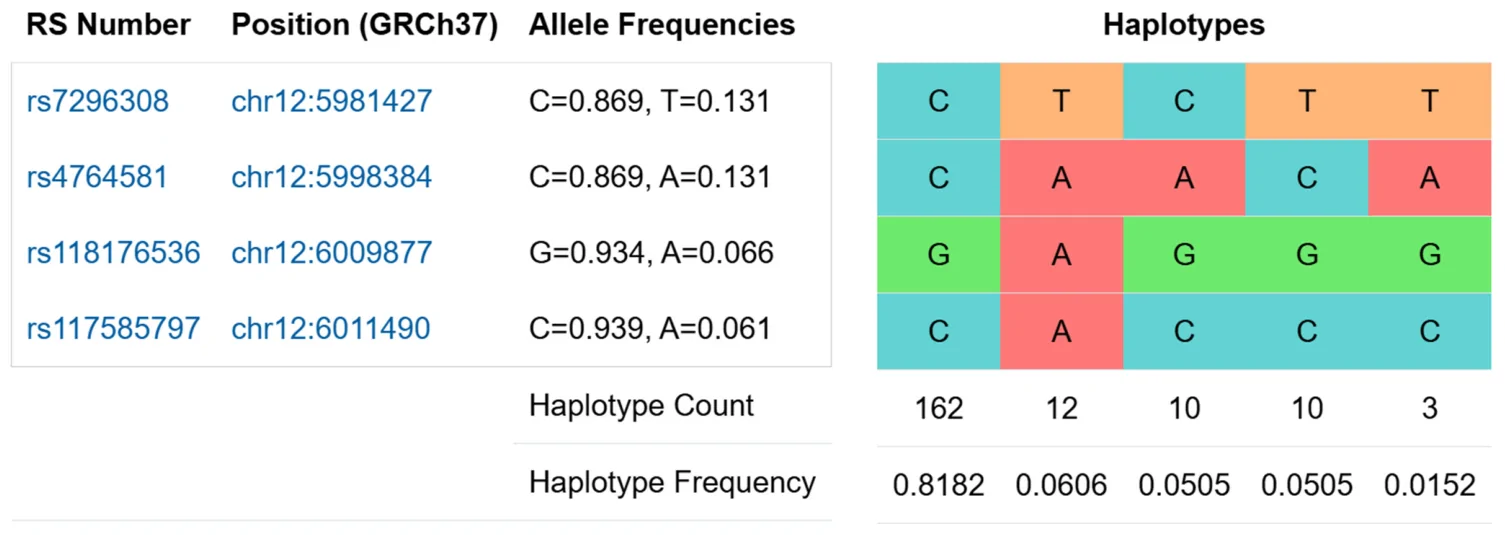
The frequency of haplotypes of the PE-associated locus rs118176536 *ANO2* (our data) and its three proxy variants linked with PE/pregnancy hypertension in women according to a replicative analysis of UK Biobank and FinnGen GWAS data the data was obtained using the LDhap program (https://ldlink.nih.gov/help#LDhap accessed on 25 August 2026) among Europeans (the 1000 Genomes Project population—Finnish in Finland (FIN)).

Analysis of (haplotypes) of the PE-associated locus rs117585797 *ANO2* (our data) and its three proxy variants (rs7296308 (T > C), rs4764581 (A > C), rs118176536 (G > A)), linked with PE/pregnancy hypertension in women according to the replication analysis of UK Biobank and FinnGen GWAS data (Table 4), showed the following (Figure 2) (the results were obtained using the LDhap program (https://ldlink.nih.gov/help#LDhap accessed on 25 August 2026) among Europeans (the 1000 Genomes Project population—Finnish in Finland (FIN))). According to the data in Figure 2, the PE-risk allele variant A rs117585797 *ANO2* is part of only one haplotype—T-A-A-A [rs7296308-rs4764581-rs118176536-rs117585797] (the frequency of this haplotype is 6.06% in the analyzed FIN population). Moreover, all the alleles included in this haplotype exhibit a unidirectional risk effect in relation to PE (Table 4).

So, as a result of replication, although we did not provide direct evidence of the association of rs117585797 *ANO2* with PE, the identified associations of several of its proxy variants with PE may be a thought-provoking argument in favor of our hypothesis that this genomic region (chromosome 12:5981427-6011490 [GRCh37]) is involved in PE. However, it should be noted that there are a number of limitations in the interpretation of the obtained results of replication analysis: two of the three PE-significant proxy variants of rs117585797 *ANO2* do not have sufficiently strong LD indicators; in the replication study, we analyzed not only the “pre-eclampsia” phenotype, but also “pregnancy hypertension”; for the replication study, we used cohorts of pregnant women without taking into account their preBMI; when evaluating the results, corrections for multiple comparisons were not taken into account. Taking into account the above, it is obvious that further associative and replicative genetic studies on this issue are needed.

### 3.2. The Proposed Functionality of the PE-Associated Locus rs117585797 ANO2 and 14 LD Polymorphisms

Analysis of the functional value of the rs117585797 *ANO2* locus associated with PE in women with a BMI < 25 and 14 variants in LD with it (r^2^ ≥ 0.2, D′ = 0.94–1 [−1]), showed that these polymorphisms, located in the intronic region of the *ANO2* gene, potentially have important regulatory relevance. Furthermore, it is very important that the potential functionality of the polymorphisms we are considering was manifested in both maternal and fetal organs, which are important in the development of PE.

First, almost all of these loci (14/15, 93.33% [with the exception of the rs4764574 locus]) were localized in DNA regions interacting with many different transcription factors (TFs) (Table S6). The interaction of DNA with the largest number of TFs was determined by such loci as rs7488107 (10 TFs), rs10459135 (9 TFs), rs11063886 (7 TFs) and rs118176536 (7 TFs). In total, these 15 polymorphic variants associated with the development of PE affect the interaction of this regulatory region of DNA (the intron of the *ANO2* gene) with 51 TFs (Table S6).

Second, a number of loci under consideration mark promoters/enhancers/“open” chromatin in the maternal organs involved in the pathophysiology of PE: the brain (rs10431363 [Epigenome ID E074:Brain Substantia Nigra]; rs7488107 [Epigenome ID E068:Brain Anterior Caudate; Epigenome ID E069: Brain Cingulate Gyrus; Epigenome ID E073:Brain_Dorsolateral_Prefrontal_Cortex]), female skeletal muscle (rs79708426 [Epigenome ID E108:Skeletal Muscle Female]), adipose tissue (rs7486935 [Epigenome ID E63:Adipose Nuclei]).

Third, individual loci under study were located in functionally active regions of the genome (promoters, enhancers, etc.) in the tissues and organs of the embryo/fetus, which may be important for the development of PE: ectoderm (rs10431363, rs6489664, rs7296308, rs79708426 and rs4764574 [Epigenome ID E011:hESC Derived CD184+ Endoderm Cultured Cells; Epigenome ID E012:hESC Derived CD56+ Ectoderm Cultured Cells]), endoderm (rs10431363, rs6489664, rs79708426, rs118176536, rs7486935 [Epigenome ID E011:hESC Derived CD184+ Endoderm Cultured Cells; Epigenome ID E012:hESC Derived CD56+ Ectoderm Cultured Cells]), neuronal progenitor cells (rs6489664, rs11063889, rs7296308, rs79708426, rs4764574, rs118176536 and rs7486935 [Epigenome ID E007:H1 Derived Neuronal Progenitor Cultured Cells]), fetal muscle (rs11063889, rs79708426, rs118176536, rs7486935 and rs117585797 [Epigenome ID E089:Fetal Muscle Trunk]; rs11063889, rs7488107, rs7296308, rs79708426, rs118176536, rs7486935 and rs117585797 [Epigenome ID E090:Fetal Muscle Leg]).

Fourth, most of the analyzed loci (11/15, 73.33%) were associated with the expression of the *ANO2* gene in peripheral blood (Table S7), alternative polyadenylation of four genes such as *NOP2*, *VAMP1*, *SCNN1A*, *IFFO1* (12/15, 80.00%) (Table S8) in various organs, including the brain (basal ganglia [rs7296308:*NOP2*] and pituitary gland [rs4764574:*IFFO1*]), which are involved in the pathophysiology of PE. Two loci (rs7488107, rs7296308) were associated with alternative splicing of the *LAG3* gene (Table S9).

Next, we studied protein interactions (PI) that were involved in the PE biology due to the functional effects of the PE-associated locus rs117585797 *ANO2* and 14 of its LD variants. According to the data presented above, these polymorphisms may exert their functional effects due to 57 different proteins such as 51 TFs (listed above) and protein products of 6 genes on which these SNPs have eQTL/sQTL/3′aQTL effects (*ANO2*, *NOP2*, *VAMP1*, *SCNN1A*, *IFFO1*, *LAG3*). Thus, in total, we examined the interactions of 57 proteins correlated with the development of PE (PI_PE_).

**Figure 3:**
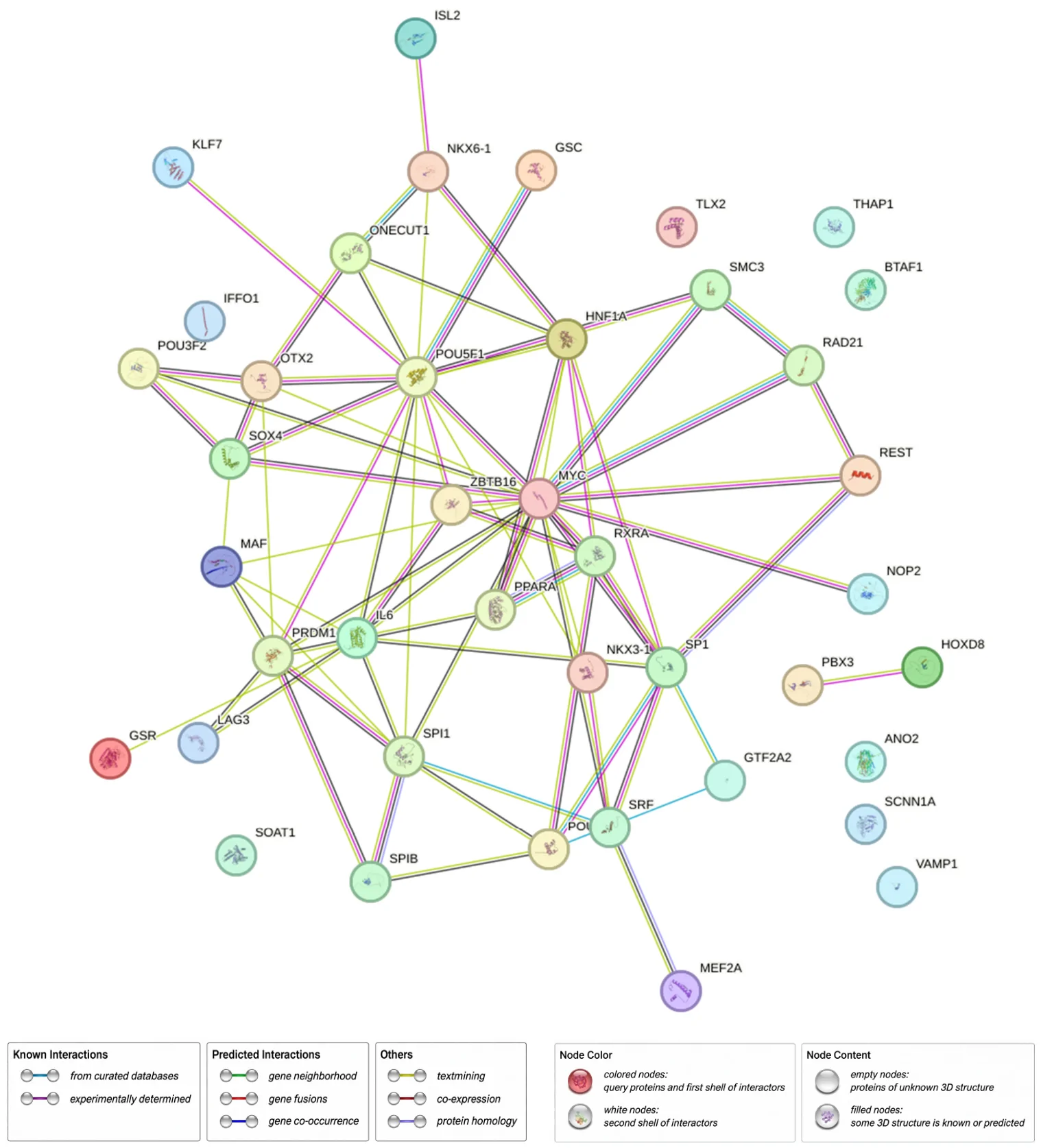
Protein interactions involved in the pathogenetics of PE due to the functional effects of the PE-associated locus rs117585797 *ANO2* and 14 SNPs in linkage disequilibrium with it (STRING data).

The analysis of biological pathways involving PI_PE_ (Figure 3, Table S10) indicates their involvement in more than 200 different pathways, among which the following were significant:

(a) regulation of gene expression (regulation of transcription by RNA polymerase II [GO:0006357, p_FDR_ = 3.6 × 10^−17^], DNA-binding transcription factor activity, RNA polymerase II-specific [GO:0000981, p_FDR_ = 1.7 × 10^−19^], cis-regulatory region sequence-specific DNA binding [GO:0000987, p_FDR_ = 1.7 × 10^−19^], regulation of miRNA transcription [GO:1902893, p_FDR_ = 1.8 × 10^−6^], histone deacetylase binding [GO:0042826, p_FDR_ = 5.6 × 10^−5^], and others);
(b) hormone-dependent processes, including estrogen-dependent ones (Steroid hormone mediated signaling pathway [GO:0043401, p_FDR_ = 0.043], regulation of hormone secretion [GO:0046883, p_FDR_ = 1.5 × 10^−4^], cellular response to steroid hormone stimulus [GO:0071383, p_FDR_ = 0.002], response to steroid hormone [GO:0048545, p_FDR_ = 0.004], ESR-mediated signaling [HSA-8939211, p_FDR_ = 0.002], estrogen-dependent gene expression [HSA-9018519, p_FDR_ = 0.002], and others);
(c) embryonic development (gastrulation [GO:0007369, p_FDR_ = 3.1 × 10^−4^], embryonic morphogenesis [GO:0048598, p_FDR_ = 5.6 × 10^−4^], embryo development [GO:0009790, p_FDR_ = 3.4 × 10^−5^], anatomical structure formation involved in morphogenesis [GO:0048646, p_FDR_ = 0.002], cell development [GO:0048468, p_FDR_ = 3.8 × 10^−5^], tissue development [GO:0009888, p_FDR_ = 6.5 × 10^−6^], cell differentiation [GO:0030154, p_FDR_ = 1.3 × 10^−5^], regulation of cell differentiation [GO:0045595, p_FDR_ = 1.4 × 10^−5^], anatomical structure morphogenesis [GO:0009653, p_FDR_ = 1.9 × 10^−8^], regulation of developmental process [GO:0050793, p_FDR_ = 1.1 × 10^−6^], artery morphogenesis [GO:0048844, p_FDR_ = 0.033], and others).

Given the BMI-dependent associations of the estrogen-related polymorphism rs117585797 *ANO2* with PE dentified in the present study, the involvement of these PI_PE_ in the processes of adipogenesis (adipogenesis [WP236, p_FDR_ = 9.6 × 10^−6^], transcription factor regulation in adipogenesis [WP3599, p_FDR_ = 0.031], regulation of lipid localization [GO:1905952, p_FDR_ = 0.035], and others) is of particular interest.

## 4. Discussion

In the investigated cohort of women (PE/control, n = 891), an association of interactions of rs117585797 *ANO2* × preBMI with PE was found (OR_A_ = 0.86). It was revealed that SNP rs117585797 *ANO2* (C > A) was linked with PE in pregnant women with a preBMI < 25 (OR_A_ = 2.69) but not in those with a preBMI ≥ 25. The PE-associated locus rs117585797 *ANO2* and 14 of its LD variants were potentially functionally significant in the PE-important organs/tissues of the mother/fetus, which are important for the pathophysiology of PE, what may confirm the hypothesis that these variants contribute to the development of PE.

In a previously conducted GWAS by Ruth et al. (a sample of 2913 European women from the TwinsUK cohort was studied) a correlation of rs117585797 *ANO2* with the estradiol levels (*p* = 1.63 × 10^−8^) was revealed; the allele variant A of this SNP was associated with a higher concentration of estradiol (β = 0.624) [33]. Thus, that the estradiol-raising allele A of the rs117585797 *ANO2* (GWAS data of Ruth et al. [33]) is associated with an increased risk of PE in women with a BMI < 25 (OR = 2.88–2.96 [our data]). Published data indicate an important role of estrogens and their metabolites in placental angiogenesis, normal trophoblast development/invasion in early pregnancy (due to the effect of estrogens on concentrations of angiogenic markers such as VEGF, s-FLT9–11, PlGF) [21,97,98,99]. In an study by Cantonwine et al. (performed in a sample of 66 pregnant women with PE and 137 pregnant women in the control group) urinary estradiol concentrations in early pregnancy were shown to be significantly (≈50%) higher than in the control group, while leveling the differences between the compared groups in late pregnancy [45]. The results of laboratory studies (including in mice) show that both a hypoxic environment and low concentrations of estradiol-17b metabolites (2-methoxyestradiol, 2-hydroxyestradiol) are necessary for the “effective” (sufficiently deep) invasion of cytotrophoblast into the uterine endometrium in early pregnancy [45,100]. At high concentration of estrogens in early pregnancy, this “condition” for the “correct” (sufficiently deep) invasion of the cytotrophoblast into the uterus will be disrupted, and this may lead to an increased risk of PE, which is consistent with our data (pronounced risk effect [OR = 2.88–2.96] of the estradiol-enhancing allele A of the rs117585797 *ANO2* in women with a BMI < 25). Our results are also in line with current concepts of the pathophysiology of PE, according to which its root cause (the “trigger” stage) is shallow trophoblast invasion, leading to inadequate remodeling of spiral arteries and subsequently causing a “negative” reaction of the mother’s body (endothelial dysfunction, imbalance between angiogenic/antiangiogenic factors, etc.), which leads to clinical manifestations of PE [1].

It should be noted that the available literature data on the concentration of estrogens (estradiol/estrone) in PE are contradictory: some studies report lower maternal estrogen concentrations [101,102,103], whereas others find no differences [101,104]. However, as a rule, the level of estrogens during pregnancy in these studies was estimated only at later stages of pregnancy [102,103]., By contrast, in the work of Cantonwine et al., which found higher concentrations of estradiol in pregnant women with PE, biological samples (urine) were analyzed in the early stages of pregnancy (5.3–15.3 weeks, median—9.8 weeks of gestation) and the authors introduced the necessary adjustments for the maternal age and BMI, gestational age, parity, race/ethnicity, use of assisted reproductive technologies [45]. There is literature data on multidirectional changes in the content of 2-methoxyestradiol in pregnant women with PE, depending on the duration of its manifestation [21,22,23,24].

Our bioinformatic analysis showed that the PE-associated locus rs117585797 *ANO2* and 14 of its LD variants were functionally significant for 6 genes (*ANO2*, *NOP2*, *VAMP1*, *SCNN1A*, *IFFO1*, *LAG3* [eQTL/sQTL/3′aQTL]) and 51 TFs (due to epigenetic DNA modifications), which were involved in a number of PE-significant biological processes (regulation of gene expression, hormone/estrogen-dependent processes, embryonic development, adipogenesis). The biological functions of the protein products of the genes listed above may help to explain the association of rs117585797 *ANO2* (and its proxy SNPs) with BMI-dependent PE risk. For example, the protein product of the *ANO2* gene, anoctamin-2, belongs to a family of calcium-activated chloride channels and is present in the smooth muscle of the human uterus [105]. In an experimental study, Bernstein et al. demonstrated the expression of ANO2 and ANO1 in human and mouse myometrium has been shown [106]. At the same time, the selective antagonism of these channels helps to weaken the spontaneous contractions of myometrial cells, and their blockade eliminates the increased level of intracellular calcium, which leads to suppression of spontaneous/induced transient internal currents [106]. The condition of the smooth muscles of the uterus is important for the normal course of pregnancy and significant changes in the membrane potential of myometrial cells, including those associated with the “work” of calcium-activated chloride channels (ANO2 and others), can lead to various disorders of pregnancy (spontaneous premature birth, and others [106]).

Another example is the protein encoded by the *SCNN1A* gene, the sodium channel epithelial 1 subunit alpha. The epithelial sodium channel serves as one of the key transporters that ensure sodium reabsorption in the distal nephron [107]. Due to this, it is important in “maintaining” the volume of extracellular fluid, inflammatory processes, regulation of blood pressure levels and the development of hypertensive reactions (arterial hypertension, etc.) [107,108]. These pathophysiological mechanisms are likely to be relevant to the development of hypertension in pregnant women, which is one of the well-known clinical symptoms of PE [8,57,60]. It is very interesting that inflammatory reactions caused by epithelial sodium channels are lipid-dependent [109]. There is evidence that the genetic variants of the *SCNN1A* are associated with the development of hypertensive disorders [107] and metabolic syndrome in women [110]. One more example is the protein encoded by the *IFFO1* gene, intermediate filament family orphan 1, which is one of the primary components of the cytoskeleton and the nuclear envelope of cells. According to the results obtained during a clinical and experimental study by Benton et al. (in the work analyzed DNA methylation in the adipose tissue of patients before and after gastric bypass surgery and related weight loss), the DNA in the region of this gene was characterized by the most pronounced hypomethylation in subcutaneous adipose tissue before weight loss compared with after weight loss [111]. Interestingly, these changes in the methylation level of the *IFFO1* gene (along with a number of other genes) detected in adipose tissue may also be characteristic of skeletal muscle in the same individuals [112].

It should be noted that a number of polymorphisms located in the region of the *ANO2* gene are GWAS-significant for such BMI/obesity-important features as “calorimetric activity” (physical activity measured during 24-h indoor calorimetry) in children (rs7307889 [113]), lysine metabolism (amino acid N-acetyl-cadaverine) in elite athletes (rs453385 [114]), and adult height (rs7967289, rs2277402, rs11611587 [115]).

In the present study, we did not identify significant association of the rs117585797 *ANO2* (C > A) polymorphism, which is associated with estradiol levels (as well as other polymorphisms involved in regulating the level of sex hormones in the body) with the risk of PE in women with preBMI ≥ 25. This may be due to the fact that overweight/obesity is a more significant independent modifier of the level of estrogens (and other sex hormones) in a woman’s body [25,26], significantly exceeding the influence of genetic determinants of estrogens in its phenotypic effect. For example, in the GWAS performed by Haas et al. in a sample of 33032 premenopausal women of European descent, the SNP heritability index for estradiol levels was only 3.9% (the authors found associations with the estradiol content of 5 SNPs) [37]. Whereas the effect of BMI on the level of estrogens has been much more pronounced and the change in the level of estradiol/estrone in a woman’s body with a change in BMI was, according to various estimates, from 11 to 101% [25,26].

Published data indicate a key role for overweight/obesity in determining the level of estrogens and other sex hormones in women [25,26,27,28,46,47]. Significantly higher estrogen levels in women with increased BMI (overweight/obese) may be explained by the following mechanisms [25,46,47,116]. First, there is a more pronounced formation of estrogens from androgens in excess adipose tissue [116]. Second, the formation of androgen precursors in the adrenal glands increases, which in turn promotes their conversion to estrogens in adipose tissue [116]. Third, the concentration of SHBG decreases [25,46], which leads to an increase in the content of free (biologically active) estrogens in the body [47]. There is strong evidence (including based on the analysis of GWAS data by Mendelian randomization [46]) of a pronounced association of BMI with estrogen/androgen levels (direct relationship) and SHBG content (reverse relationship) [25]. Thus, in the work of Stanczyk et al. (data on 1187 women were analyzed) it was shown that and estradiol levels in women with a BMI = 25–29.9 (30 pg/mL and 9 pg/mL) and a BMI ≥ 30 (37 pg/mL and 13 pg/mL) were higher (on 11–37% and 29–86% respectively) than in women with a BMI < 25 (27 pg/mL and 7 pg/mL) [25]. It is very interesting that an increase of the BMI in women determines a significant increase in concentrations of free [↑101%] and total [↑45–68%] estradiol, estrone [↑21–34%], testosterone [↑35%], and a decrease in SHBG levels [↓6–35%] [26], and a decrease in BMI, conversely, leads to a “normalization” of the level of sex hormones (a decrease of estrogen/testosterone levels, an increase of SHBG concentration) [27,28]. This may be of considerable practical importance for the prevention of BMI-dependent diseases, including PE.

The role of increased BMI (overweight and obesity) as a risk factor for PE has been demonstrated in numerous studies [7,9,12,17,19]. Thus, in the work of Durst et al. (a sample of 10196 pregnant women was studied), it was shown that overweight (OR = 1.4) and obesity (OR = 2.0) significantly increase the risk of severe PE [13]. Venkatesh et al., using Mendelian randomization of GWAS data in a sample of 257193 women of European ancestry, confirmed the causal genetic relationship of BMI (OR = 2.09 per kilogram of BMI) and visceral adipose tissue (VAT) (OR = 3.08 per kilogram of VAT) with PE [15]. A significant association of overweight (OR = 1.71) and obesity (OR = 2.48) with the risk of PE was also shown in the meta-analysis by He et al., conducted on the basis of data from 19 studies on this topic [14]. A comprehensive analysis (meta-analysis and Mendelian randomization) performed by Sun et al. revealed a significant association between maternal overweight/obesity before pregnancy and an increased risk of PE (OR = 1.78 for overweight and OR = 3.46 for obesity) [16]. The authors also found that each 1% increase in genetically predicted obesity rates (BMI, hip circumference, total body fat mass, body fat mass, body fat percentage) was associated with an increased risk of developing PE [16]. Possible mechanisms mediating the risky effect of overweight/obesity on the development of PE include endothelial dysfunction, dyslipidemia, insulin resistance, chronic inflammation, leptin action, oxidative stress, hyperestrogenism, among others [7,8,9,10,11,12,13,14,15,16,17,18,19].

PreBMI (*p* = 0.008) and obesity (OR = 2.94, *p* = 0.0005) were also risk factors for PE in the sample of pregnant women which we studied [57]. In a previous genetic study of PE in the same sample of patients/controls with different preBMI by Abramova et al. associations of GWAS-significant hypertension/blood pressure loci with PE (rs167479 *RGL3* and rs805303 *BAG6*) in women with preBMI ≥ 25 and the absence of these associations in women with preBMI < 25 were shown [60]. It should be noted that the differences between our results (the presence of an association at the GWAS locus, significant for estradiol levels, with PE in women with preBMI < 25 and its absence in women with preBMI ≥ 25) and the data of Abramova et al. (associations with PE of loci that are GWAS-significant for hypertension and blood pressure in women with preBMI ≥ 25 and their absence in women with preBMI < 25) [60] may be due to the fact that high preBMI (overweight/obesity) and hypertension/blood pressure are comorbid [19,117,118], unidirectional factors in relation to PE (increase the risk of PE [13,14,15,19,119,120]), having “common” genetic determinants (as confirmed by GWAS and Mendelian randomization studies) [6,15,16,113,121,122,123,124,125,126] (for example, the indicators of genetic correlations between systolic/diastolic blood pressure, hypertension and PE were r_g_ = 0.3–0.4 [6]). Thus, the combined effect of these factors (and their genetic determinants) may enhance their phenotypic risk effect in PE (in women with preBMI ≥ 25), as found in the study by Abramova et al. [60]. At the same time, the influence of genetic determinants on the level of sex hormones (estradiol) is extremely weak [37] and may be phenotypically “visible” in the development of PE in women who lack more significant factors affecting the level of sex hormones in the body (overweight/obesity [25,26,46]), which we have documented in our work in women with a preBMI ≥ 25. The modifying effect of BMI on the nature of the SNP association of a number of candidate genes in the formation of BMI-dependent diseases such as breast cancer (matrix metalloproteinases were studied) [78], knee osteoarthritis (GWAS loci significant for the disease were considered) [84], and uterine fibroids (GWAS variants significant for SHBG levels were studied) [68] was shown by our team in previous studies in the same population (Europeans of Central Russia).

This study has a number of limitations: (a) our results require replication in other European populations outside Central Russia; (b) the association with PE was found for a rare variant with a very small number of carriers (MAF in the studied cohort preBMI < 25 is 2.72%); the resulting estimate is unstable (at OR = 2.69, 95%CI varies from 1.26 to 5.72), and highly susceptible to sparse-data bias.

## 5. Conclusions

In the present study, BMI—specific link between GWAS-significant for estradiol levels polymorphism rs117585797 *ANO2* (C > A) and PE was demonstrated: this SNP was associated with PE in preBMI < 25 pregnant women, whereas no association was observed in women with preBMI ≥ 25.

## Figures and Tables

**Table 1:** The phenotypic/clinical characteristics of the subjects as a whole.

Parameters	Pre-Eclampsia, $\bar{X}$ ± SD/% (n)	Controls, $\bar{X}$ ± SD/% (n)
*N*	431	460
Age, years (min–max)	27.36 ± 5.06 (17–43)	26.58 ± 4.94 (16–42)
Pre-pregnancy BMI, kg/m^2^	24.85 ± 5.23	23.56 ± 3.61
Gestational age at recruitment (mean, weeks) (min–max)	36.84 ± 3.32 (28–38)	38.72 ± 1.54 (37–40)
Gestational age at onset of PE (mean, weeks)	34.56 ± 3.67	-
Gestational age at onset of PE, early onset/late onset	14.15/85.85 (61/370)	-
Severity of PE, severe/mild	16.70/83.30 (72/359)	-
Fetal growth restriction	19.26 (83)	-

**Table 2:** Phenotypic characteristics of the study participants (preBMI < 25 and preBMI ≥ 25).

Parameters	preBMI ≥ 25			preBMI < 25		
	Pre-Eclampsia $\bar{X}$ ± SD/% (n)	Controls $\bar{X}$ ± SD/% (n)	*p*	Pre-Eclampsia $\bar{X}$ ± SD/% (n)	Controls $\bar{X}$	*p*
*N*	152	147	-	279	313	-
Age, years (min–max)	29.03 ± 5.07 (18–42)	27.07 ± 5.29 (18–41)	**0.001**	26.49 ± 4.82 (17–43)	26.35 ± 4.75 (16–42)	0.70
Pre-pregnancy BMI, kg/m^2^	30.49 ± 4.50	27.80 ± 2.42	**0.0001**	21.73 ± 1.87	21.62 ± 2.01	0.34
Family history of PE	25.00 (38)	13.61 (20)	**0.02**	22.94 (64)	11.50 (36)	0.001
Smoker (yes)	48.68 (74)	53.06 (78)	0.52	44.44 (124)	52.40 (164)	0.06
Alcohol consumption (yes)	77.63 (118)	83.67 (123)	0.24	74.19 (207)	77.32 (242)	0.43
Pre-pregnancy blood pressure (BP)						
Systolic BP, mm Hg	113.80 ±9.62	113.68 ± 6.51	0.75	111.93 ± 9.97	110.60 ± 9.27	0.09
Diastolic BP, mm Hg	72.27 ± 6.42	72.80 ± 6.27	0.51	71.66 ± 5.34	71.04 ± 4.25	0.52
Mean BP, mm Hg	86.12 ± 7.28	86.60 ± 6.32	0.26	85.22 ± 8.15	84.89 ± 7.64	0.15
Pulse BP, mm Hg	40.30 ± 4.90	39.96 ± 3.71	0.13	39.63 ± 4.34	38.54 ± 4.22	0.10
Age at menarche and menstrual cycle						
Age at menarche, years	12.16 ± 1.16	12.38 ± 1.00	0.51	12.70 ± 0.91	12.66 ± 1.08	0.17
Duration of bleeding menstrual (mean, days)	4.86 ± 1.31	5.02 ± 0.75	0.19	4.91 ± 1.08	4.97 ± 0.76	0.49
Menstrual cycle length (mean, days)	27.43 ± 3.12	28.14 ± 1.14	0.17	28.16 ± 2.65	28.45 ± 1.85	0.62
Reproductive status						
First pregnancy	35.53 (54)	37.41 (55)	0.83	41.58 (116)	44.41 (139)	0.54
No of gravidity (mean)	2.13 ± 1.86	1.40 ± 1.68	**0.03**	1.09 ± 1.31	1.01 ± 1.32	0.39
No of births (mean)	0.81 ± 0.88	0.79 ± 0.86	0.72	0.40 ± 0.53	0.41 ± 0.61	0.67
No of spontaneous abortions (mean)	0.27 ± 0.51	0.11 ± 0.33	**0.009**	0.21 ± 0.45	0.15 ± 0.41	0.09
No of induced abortions (mean)	0.96 ± 1.31	0.46 ± 0.86	**0.005**	0.43 ± 0.74	0.42 ± 0.80	0.41
No of stillbirths	0.07 ± 0.24	0.02 ± 0.13	0.09	0.03 ± 0.16	0.01 ± 0.12	0.08
Somatic pathologies						
Cardiovascular	17.76 (27)	10.88 (16)	0.13	11.47 (32)	9.27 (29)	0.46
Kidney	7.24 (11)	4.76 (7)	0.51	4.31 (12)	2.87 (9)	0.47
Endocrine	3.95 (6)	3.40 (5)	1.00	2.87 (8)	0.96 (3)	0.16
Gastrointestinal	3.29 (5)	1.36 (2)	0.47	1.79 (5)	3.51 (11)	0.30

Note: *p* values < 0.05 are shown in bold.

**Table 3:** Associations of the SNP-BMI interactions with pre-eclampsia in total cohorts of patient/control and associations of the studied polymorphisms with pre-eclampsia among preBMI < 25 and preBMI ≥ 25 female.

Chr	SNP	Minor Allele	Gene	n	SNP-BMI Interaction					Female with preBMI < 25 *					Female with preBMI ≥ 25 *			
					OR	95%CI		P	n	OR	95%CI		P	n	OR	95%CI		P
						L95	U95				L95	U95				L95	U95	
1	rs17496332	G	*PRMT6*	865	0.99	0.95	1.04	0.900	569	1.01	0.80	1.28	0.925	296	1.03	0.73	1.45	0.855
2	rs780093	T	*GCKR*	889	1.01	0.96	1.05	0.728	591	0.99	0.78	1.25	0.924	298	1.03	0.73	1.46	0.875
2	rs10454142	C	*PPP1R21/KLRAQ*	880	0.99	0.94	1.03	0.577	586	1.11	0.87	1.41	0.412	294	1.30	0.88	1.92	0.184
7	rs3779195	A	*BAIAP2L1*	878	1.01	0.95	1.06	0.753	586	1.07	0.79	1.43	0.677	292	1.16	0.74	1.82	0.524
7	rs148982377	C	*ZNF789*	888	0.99	0.89	1.11	0.958	590	0.68	0.39	1.20	0.180	298	0.51	0.23	1.16	0.107
7	rs34670419	T	*ZKSCAN5*	889	0.99	0.91	1.09	0.968	590	0.95	0.52	1.73	0.862	299	0.61	0.27	1.39	0.239
8	rs440837	G	*ZBTB10*	869	1.03	0.98	1.08	0.240	578	0.92	0.70	1.22	0.575	291	1.16	0.75	1.82	0.506
10	rs7910927	T	*JMJD1C*	883	1.01	0.96	1.05	0.685	586	0.84	0.67	1.05	0.118	297	0.86	0.60	1.25	0.434
11	rs11031002	A	*FSHB*	888	0.97	0.90	1.03	0.351	590	0.99	0.71	1.38	0.936	298	0.90	0.54	1.49	0.674
11	rs11031005	C	*FSHB*	890	0.99	0.93	1.04	0.643	591	0.96	0.69	1.33	0.799	299	0.98	0.58	1.66	0.936
11	rs112295236	G	*SLC22A10*	890	1.05	0.94	1.17	0.394	591	0.83	0.48	1.43	0.505	299	0.93	0.41	2.10	0.855
12	rs117585797	A	*ANO2*	888	**0.86**	**0.76**	**0.96**	**0.019**	589	**2.69**	**1.26**	**5.72**	**0.010**	299	0.48	0.16	1.49	0.204
12	rs4149056	C	*SLCO1B1*	838	0.97	0.93	1.02	0.287	556	1.35	1.02	1.78	0.038	282	0.98	0.63	1.51	0.918
15	rs8023580	C	*NR2F2*	882	0.98	0.94	1.03	0.549	588	1.23	0.97	1.58	0.091	294	0.98	0.67	1.44	0.937
16	rs117145500	C	*CHD9*	886	1.00	0.94	1.07	0.958	587	0.98	0.66	1.45	0.921	299	0.97	0.53	1.77	0.911
17	rs12150660	T	*SHBG*	888	1.00	0.95	1.05	0.953	591	0.82	0.57	1.19	0.298	297	0.80	0.45	1.42	0.439
17	rs727428	T	*SHBG*	890	1.02	0.98	1.07	0.277	591	0.68	0.47	1.00	0.047	299	1.32	0.72	2.42	0.374
17	rs1641549	T	*TP53*	886	0.96	0.92	1.02	0.237	589	1.09	0.77	1.56	0.615	297	0.62	0.34	1.13	0.117

Note: OR–odds ratio; 95% CI–95% confidence interval; *—results were obtained with Firth’s penalized regression for the additive model after adjusting for covariates; P_permt_ values < 0.025 are shown in bold; Statistically significant values are shown in bold.

**Table 4:** The results of replication at association of proxy variants at the PE-causal locus rs117585797 *ANO2* with the PE risk and the PE-significant trait (pregnancy hypertension) using UK Biobank and FinnGen GWAS data.

SNP	r^2^/D′ *	Effect Allele (Risk Allele)	Gestational Hypertension/Pre-Eclampsia (UK Biobank GWAS Data) Case-1829 Control-243665	Pre-Eclampsia or Eclampsia (Meta-Analysis of UK Biobank and FinnGen GWAS Data) Case-8509 Control-437855	Pregnancy Hypertension	
					(FinnGen GWAS Data) Case-16417 Control-213893	(Meta-Analysis of UK Biobank and FinnGen GWAS Data) Case-18134 Control-439699
rs7296308 (T > C)	0.43/−1.00	C (T)		β = −0.32, *p* = 0.033	β = −0.23, *p* = 0.044	β = −0.24, *p* = 0.019
rs4764581 (A > C)	0.43/−1.00	C (A)			β = −0.35, *p* = 0.048	
rs118176536 (G > A)	0.92/1.00	A (A)	OR = 1.28, *p* = 0.037			

Note: *—the results were obtained using the LDmatrix program (https://ldlink.nih.gov/ldmatrix accessed on 25 August 2026) among Europeans (the 1000 Genomes Project population—Finnish in Finland (FIN)).

## Data Availability

From the corresponding author upon reasonable request. The data generated in the present study are available from the corresponding author upon reasonable request.

## Supplementary Materials

The following supporting information can be downloaded at: https://www.mdpi.com/article/10.3390/life16091472/s1.

## Abbreviations

The following abbreviations are used in this manuscript:

BMI	Body mass index
PE	Pre-eclampsia
SNP	Single nucleotide polymorphism
GWAS	Genome-wide association studies
SHBG	Sex hormone-binding globulin
LD	Linkage disequilibrium
