# Supplementary material for: BMI-Dependent Associations of the Estradiol-Linked Polymorphism rs117585797 of the Anoctamin-2 Gene with Pre-Eclampsia
Source: Life (Basel). 2026 Sep 3;16(9):1472. doi: 10.3390/life16091472 (PMC13608154; doi:10.3390/life16091472)
Supplement: Supplementary file 1 [file life-16-01472-s001.zip › Supplementary Table S1.pdf]

Table S1. The GWAS data about associations of the studied candidate genes polymorphisms with the level of sex hormones

SNP	Position (chr: hg38)	Phenotype	Association (significance) (affected allele)	Reference	Population
rs17496332	1: 107003753	SHBG	β=-0.028 ($p=1\times10^{-11}$) (A)	Coviello A.D. et al., 2012	Europeans
rs780093	2: 27519736	SHBG	β=-0.032 ($p=2\times10^{-16}$) (T)	Coviello A.D. et al., 2012	Europeans
rs10454142	2: 48419260	SHBG	β=0.026 ($p=1\times10^{-7}$) (T)	Coviello AD et al., 2012	Europeans
rs3779195	7: 98364050	SHBG	β=-0.033 ($p=3\times10^{-8}$) (A)	Coviello A.D. et al., 2012	Europeans
		SHBG (women, pre-menopause)	β=-2.41 ($p=9\times10^{-09}$) (A)	Harrison S. et al., 2021	Europeans
rs148982377	7: 99477415	DHEAS	β = -0.255 ($p = 1.82 \times 10^{-14}$) (C)	Ruth K.S. et al., 2016	Europeans
		Bioavailable Testosterone (premenopausal women)	β = -0.05 ($p = 4.29 \times 10^{-13}$) (C)	Harrison S. et al., 2021	Europeans
		Androsterone sulfate levels	β = -1.50 ($p = 1.00 \times 10^{-337}$) (C)	Chen Y. et al., 2023	Europeans
		Epiandrosterone sulfate levels	β = -1.32 ($p = 4.00 \times 10^{-270}$) (C)	Chen Y. et al., 2023	Europeans
		Androstenediol (3beta,17beta) disulfate (1) levels	β = -0.84 ($p = 5.00 \times 10^{-116}$) (C)	Chen Y. et al., 2023	Europeans
		Androstenediol (3alpha, 17alpha) monosulfate (3) levels	β = -0.31 ($p = 5.00 \times 10^{-16}$) (C)	Chen Y. et al., 2023	Europeans
rs34670419	7: 99533211	Progesterone	β = -0.346 ($p = 6.09 \times 10^{-14}$) (T)	Ruth K.S. et al., 2016	Europeans
		Cortisol/DHEAS ratio	β = 0.721 (p = 2.35 × 10-8) (T)	Wood A.R. et al., 2013	Europeans
		DHEAS	β = -0.780 ($p = 2.07 \times 10^{-9}$) (T)	Wood A.R. et al., 2013	Europeans
		4-androsten-3beta,17beta-diol disulfate (1) levels	β = -0.39 ($p = 4.00 \times 10^{-28}$) (T)	Hysi P.G. et al., 2022	Europeans
		4-androsten-3beta,17beta-diol monosulfate (1) levels	β = -0.47 ($p = 1.00 \times 10^{-41}$) (T)	Hysi P.G. et al., 2022	Europeans
		4-androsten-3beta,17beta-diol monosulfate (2) levels	β = -0.46 ($p = 2.00 \times 10^{-32}$) (T)	Hysi P.G. et al., 2022	Europeans
rs440837	8: 80549739	SHBG	β=-0.030 ($p=3\times10^{-9}$) (A)	Coviello A.D. et al., 2012	Europeans
		SHBG (women, post-menopause)	β=1.43 ($p=1\times10^{-12}$) (G)	Harrison S. et al., 2021	Europeans
		SHBG (men)	β=0.57 ($p=8\times10^{-9}$) (G)	Harrison S. et al., 2021	Europeans

rs7910927	10: 63379150	SHBG	β=-0.048 ($p=6\times10^{-35}$) (T)	Coviello A.D. et al., 2012	Europeans
rs11031002	11: 30193714	LH	β = 0.221 ($p = 3.94 \times 10^{-9}$) (A)	Ruth K.S. et al., 2016	Europeans
		Serum levels of protein CGA;FSHB	β = -0.162 ($p = 5.00 \times 10^{-20}$) (A)	Gudjonsson A. et al., 2022	Europeans
rs11031005	11: 30204809	FSH	β = -0.232 ($p = 1.74 \times 10^{-8}$) (C)	Ruth K.S. et al., 2016	Europeans
		Total testosterone	β = 0.033 ($p = 7.2 \times 10^{-17}$) (C)	Ruth K.S. et al., 2020	Europeans
		Bioavailable testosterone	β = 0.023 ($p = 1.5 \times 10^{-10}$) (C)	Ruth K.S. et al., 2020	Europeans
		Total testosterone	β = 0.027 ($p = 7.22\times 10^{-9}$) (C)	Haas C.B. et al., 2022	Europeans
		Free testosterone levels (women)	β =-0.031 ($p = 2.00 \times 10^{-10}$) (T)	Leinonen J.T. et al., 2023	Europeans
		FAI (women)	β =-0.031 ($p = 4.00 \times 10^{-10}$) (T)	Leinonen J.T. et al., 2023	Europeans
rs112295236	11: 63147874	Progesterone	β = 0.255 ($p = 7.68 \times 10^{-12}$) (G)	Ruth K.S. et al., 2016	Europeans
		Bioavailable testosterone (in men and women combined)	β = 0.031 ($p = 2.3 \times 10^{-14}$) (G)	Ruth K.S. et al., 2020	Europeans
		Pregnanediol-3-glucuronide levels (men)	β = 0.52 ($p = 3.0 \times 10^{-14}$) (G)	Yin X. et al., 2022	Europeans
rs117585797	12: 5902324	Estradiol	β = 0.624 ($p = 1.63 \times 10^{-8}$) (A)	Ruth K.S. et al., 2016	Europeans
rs4149056	12: 21178615	SHBG	β=0.029 ($p=2\times10^{-8}$) (T)	Coviello A.D. et al., 2012	Europeans
		low testosterone levels (men)	OR=1.14 ($p=7\times10^{-16}$) (C)	Fantus R.J. et al., 2021	Europeans
		testosterone (women)	β=0.028 ($p=5\times10^{-10}$) (C)	Haas C.B. et al., 2022	Europeans
		SHBG (women)	β=-0.065 ($p =5\times10^{-48}$) (C)	Haas C.B. et al., 2022	Europeans
		SHBG (women, pre-menopause)	β=-0.062 ($p=8\times10^{-11}$) (C)	Haas C.B. et al., 2022	Europeans
		SHBG (women, post-menopause)	β=-0.079 ($p=7\times10^{-34}$) (C)	Haas C.B. et al., 2022	Europeans
		bioavailable testosterone (women)	β=0.02 ($p=2\times10^{-16}$) (C)	Harrison S. et al., 2021	Europeans
		SHBG (men)	β=-1.23 ($p=7\times10^{-29}$) (C)	Harrison S. et al., 2021	Europeans
		SHBG (women)	β=0.030 ($p=1\times10^{-73}$) (T)	Ruth K.S. et al., 2020	Europeans
		SHBG (men)	β=0.032 ($p=6\times10^{-99}$) (T)	Ruth K.S. et al., 2020	Europeans
		total testosterone (women)	β=-0.029 ($p=1\times10^{-14}$) (T)	Ruth K.S. et al., 2020	Europeans
		bioavailable testosterone	β=-0.043 ($p=3\times10^{-35}$) (T)	Ruth K.S. et al., 2020	Europeans

		(women)			
		total testosterone (men)	β=0.054 ($p=1\times10^{-39}$) (T)	Ruth K.S. et al., 2020	Europeans
rs8023580	15: 96165062	SHBG	β=-0.03 ($p=8\times10^{-12}$) (T)	Coviello A.D. et al., 2012	Europeans
		low testosterone levels (men)	OR=1.13 ($p=1\times10^{-19}$) (T)	Fantus R.J. et al., 2021	Europeans
rs117145500	16: 52913718	FAI	β = -0.276 ($p = 1.50\times10^{-8}$) (C)	Ruth K.S. et al., 2016	Europeans
rs12150660	17: 7618597	SHBG	β=0.103 ($p=2\times10^{-106}$) (T)	Coviello A.D. et al., 2012	Europeans
		SHBG (women)	β=6.14 ($p=1\times10^{-300}$) (T)	Harrison S. et al., 2021	Europeans
		SHBG (men)	β=3.9 ($p=2\times10^{-75}$) (T)	Ohlsson C. et al., 2011	Europeans
		total testosterone (men)	β=31.8 ($p=1\times10^{-41}$) (T)	Ohlsson C. et al., 2011	Europeans
rs727428	17: 7634474	SHBG	β=-0.126 ($p=2.09\times10^{-16}$)(T)	Prescott J. et al., 2012	Europeans
		bioavailable testosterone	β=0.095 ($p=8.3\times10^{-309}$) (T)	Ruth K.S. et al., 2020	Europeans
		oestradiol	β=-0.041 ($p=2.10\times10^{-10}$)(T)	Haas C.B. et al., 2022	Europeans
		free testosterone (women)	β=-0.001 ($p=5.00\times10^{-21}$)(C)	Harrison S. et al., 2021	Europeans
		SHBG (premenopausal women)	β=7.25 ($p=5.43\times10^{-109}$) (C)	Harrison S. et al., 2021	Europeans
		bioavailable testosterone (postmenopausal women)	β=-0.019 ($p=3.09\times10^{-25}$)(C)	Harrison S. et al., 2021	Europeans
		bioavailable testosterone (women)	β=-0.021 ($p=1.03\times10^{-48}$)(C)	Harrison S. et al., 2021	Europeans
rs1641549	17: 7671457	SHBG	β=-0.127 ($p=1.21\times10^{-15}$)(T)	Ruth K.S. et al., 2016	Europeans

Note: Chr, chromosome; SHBG, sex hormone binding globulin; DHEAS, dehydroepiandrosterone sulphate; FAI, free androgen index ((testosterone/SHBG) × 100); FSH, follicle-stimulating hormone; LH, luteinizing hormone.
