# Supplementary material for: BMI-Dependent Associations of the Estradiol-Linked Polymorphism rs117585797 of the Anoctamin-2 Gene with Pre-Eclampsia
Source: Life (Basel). 2026 Sep 3;16(9):1472. doi: 10.3390/life16091472 (PMC13608154; doi:10.3390/life16091472)
Supplement: Supplementary file 1 [file life-16-01472-s001.zip › Supplementary Table S3.pdf]

Table S3. The allele and genotype frequencies of the studied SNPs in the pre-eclampsia and control groups with preBMI<25

Chr	SNP	Minor allele	Major allele	Minor allele frequency	Genotype distribution*	H_o	H_e	P_{HWE}
Pre-eclampsia patients (n=279)								
1	rs17496332	G	A	0.372	36/120/102	0.465	0.467	1.000
2	rs780093	T	C	0.396	44/133/102	0.477	0.478	1.000
2	rs10454142	C	T	0.351	40/114/122	0.413	0.456	0.115
7	rs3779195	A	T	0.186	11/81/185	0.292	0.303	0.553
7	rs148982377	C	T	0.038	0/21/258	0.075	0.072	1.000
7	rs34670419	T	G	0.038	0/21/258	0.075	0.072	1.000
8	rs440837	G	A	0.233	13/100/157	0.370	0.358	0.733
10	rs7910927	T	G	0.461	63/131/85	0.470	0.497	0.399
11	rs11031002	A	T	0.128	8/55/215	0.198	0.223	0.098
11	rs11031005	C	T	0.133	6/62/211	0.222	0.230	0.600
11	rs112295236	G	C	0.043	0/24/255	0.086	0.082	1.000
12	rs117585797	A	C	0.041	1/21/257	0.075	0.079	0.377
12	rs4149056	C	T	0.254	18/90/140	0.363	0.379	0.504
15	rs8023580	C	T	0.327	32/118/128	0.425	0.440	0.586
16	rs117145500	C	A	0.089	3/43/230	0.156	0.162	0.458
17	rs12150660	T	G	0.215	16/88/175	0.315	0.338	0.287
17	rs727428	T	C	0.384	40/134/105	0.480	0.473	0.899
17	rs1641549	T	C	0.275	18/117/143	0.421	0.399	0.452
Control group (n=313)								
1	rs17496332	G	A	0.369	44/142/126	0.455	0.466	0.716
2	rs780093	T	C	0.399	50/150/113	0.479	0.480	1.000
2	rs10454142	C	T	0.330	30/145/136	0.466	0.442	0.371
7	rs3779195	A	T	0.174	10/88/212	0.284	0.288	0.843

7	rs148982377	C	T	0.056	0/35/277	0.112	0.106	0.610
7	rs34670419	T	G	0.042	0/26/286	0.083	0.080	1.000
8	rs440837	G	A	0.249	14/126/169	0.408	0.374	0.130
10	rs7910927	T	G	0.507	82/148/78	0.481	0.500	0.495
11	rs11031002	A	T	0.128	5/70/238	0.224	0.223	1.000
11	rs11031005	C	T	0.136	7/71/235	0.227	0.235	0.478
11	rs112295236	G	C	0.053	1/31/281	0.099	0.100	0.589
12	rs117585797	A	C	0.014	0/9/302	0.029	0.029	1.000
12	rs4149056	C	T	0.206	14/99/196	0.320	0.327	0.728
15	rs8023580	C	T	0.285	29/119/163	0.383	0.407	0.329
16	rs117145500	C	A	0.091	4/49/259	0.157	0.166	0.305
17	rs12150660	T	G	0.249	23/110/180	0.351	0.374	0.291
17	rs727428	T	C	0.399	54/142/117	0.454	0.480	0.347
17	rs1641549	T	C	0.250	27/102/183	0.327	0.375	0.024

Note: * minor allele homozygotes / heterozygotes / major allele homozygotes
