# Supplementary material for: BMI-Dependent Associations of the Estradiol-Linked Polymorphism rs117585797 of the Anoctamin-2 Gene with Pre-Eclampsia
Source: Life (Basel). 2026 Sep 3;16(9):1472. doi: 10.3390/life16091472 (PMC13608154; doi:10.3390/life16091472)
Supplement: Supplementary file 1 [file life-16-01472-s001.zip › Supplementary Table S4.pdf]

Table S4. The allele and genotype frequencies of the studied SNPs in the pre-eclampsia and control groups with preBMI≥25

Chr	SNP	Minor allele	Major allele	Minor allele frequency	Genotype distribution*	H_o	H_e	P_{HWE}
Pre-eclampsia patients (n=152)								
1	rs17496332	G	A	0.377	27/59/64	0.393	0.470	0.055
2	rs780093	T	C	0.404	29/64/58	0.424	0.482	0.175
2	rs10454142	C	T	0.311	14/64/70	0.432	0.428	1.000
7	rs3779195	A	T	0.170	6/38/103	0.259	0.282	0.375
7	rs148982377	C	T	0.036	0/11/140	0.073	0.070	1.000
7	rs34670419	T	G	0.033	1/8/143	0.053	0.064	0.142
8	rs440837	G	A	0.231	7/54/86	0.367	0.356	0.819
10	rs7910927	T	G	0.470	28/87/37	0.572	0.498	0.076
11	rs11031002	A	T	0.128	1/37/114	0.243	0.224	0.470
11	rs11031005	C	T	0.135	1/39/112	0.257	0.233	0.313
11	rs112295236	G	C	0.046	0/14/138	0.092	0.088	1.000
12	rs117585797	A	C	0.020	0/6/146	0.039	0.039	1.000
12	rs4149056	C	T	0.215	9/41/87	0.299	0.338	0.204
15	rs8023580	C	T	0.273	12/58/80	0.387	0.397	0.837
16	rs117145500	C	A	0.105	1/30/121	0.197	0.188	1.000
17	rs12150660	T	G	0.258	12/54/85	0.358	0.383	0.400
17	rs727428	T	C	0.375	18/78/56	0.513	0.469	0.300
17	rs1641549	T	C	0.183	4/47/99	0.313	0.299	0.786
Control group (n=147)								
1	rs17496332	G	A	0.366	22/63/61	0.432	0.464	0.378
2	rs780093	T	C	0.415	25/72/50	0.490	0.486	1.000
2	rs10454142	C	T	0.250	9/55/82	0.377	0.375	1.000
7	rs3779195	A	T	0.166	8/32/105	0.221	0.276	0.029

7	rs148982377	C	T	0.061	1/16/130	0.109	0.115	0.425
7	rs34670419	T	G	0.044	1/11/135	0.075	0.085	0.242
8	rs440837	G	A	0.191	4/47/93	0.326	0.309	0.786
10	rs7910927	T	G	0.510	36/76/33	0.524	0.500	0.620
11	rs11031002	A	T	0.140	3/35/108	0.240	0.241	1.000
11	rs11031005	C	T	0.136	2/36/109	0.245	0.235	1.000
11	rs112295236	G	C	0.058	0/17/130	0.116	0.109	1.000
12	rs117585797	A	C	0.034	0/10/137	0.068	0.066	1.000
12	rs4149056	C	T	0.217	5/53/87	0.366	0.340	0.469
15	rs8023580	C	T	0.260	13/49/82	0.340	0.385	0.192
16	rs117145500	C	A	0.102	0/30/117	0.204	0.183	0.364
17	rs12150660	T	G	0.271	11/57/78	0.390	0.395	0.837
17	rs727428	T	C	0.357	13/79/55	0.537	0.459	0.049
17	rs1641549	T	C	0.208	7/47/93	0.320	0.329	0.801

Note: * minor allele homozygotes / heterozygotes / major allele homozygotes
