# Supplementary material for: BMI-Dependent Associations of the Estradiol-Linked Polymorphism rs117585797 of the Anoctamin-2 Gene with Pre-Eclampsia
Source: Life (Basel). 2026 Sep 3;16(9):1472. doi: 10.3390/life16091472 (PMC13608154; doi:10.3390/life16091472)
Supplement: Supplementary file 1 [file life-16-01472-s001.zip › Supplementary Table S5.pdf]

Table S5. Associations of the gene polymorphism haplotypes correlated with sex hormone levels with pre-eclampsia among preBMI<25 and preBMI≥25 female

Haplotypes	Frequency		OR	P
	Pre-eclampsia	Controls		
female with preBMI<25				
rs148982377 *ZNF789* - rs34670419 *ZKSCAN5*				
CT	0.034	0.040	0.82	0.598
CG	0.004	0.016	0.24	0.048
TG	0.962	0.943	1.47	0.170
rs11031002-rs11031005 *FSHB*				
AC	0.120	0.122	0.96	0.827
TC	0.013	0.014	0.94	0.792
TT	0.867	0.863	1.00	0.931
female with preBMI<25				
rs148982377 *ZNF789* - rs34670419 *ZKSCAN5*				
CT	0.027	0.044	0.56	0.214
CG	0.010	0.017	0.40	0.284
TG	0.963	0.939	1.86	0.123
rs11031002-rs11031005 *FSHB*				
AC	0.115	0.127	0.93	0.810
TC	0.020	0.010	1.31	0.763
AT	0.013	0.014	0.64	0.557
TT	0.852	0.849	1.09	0.744

Note: The results were obtained by the logistic regression analysis with adjustment for covariates; OR, odds ratio; p, significance level
